# Optimizing novel penetration enhancing hybridized vesicles for augmenting the *in-vivo* effect of an anti-glaucoma drug

**DOI:** 10.1080/10717544.2016.1233588

**Published:** 2017-02-03

**Authors:** Sarah S. Naguib, Rania M. Hathout, Samar Mansour

**Affiliations:** 1 Department of Pharmaceutical Technology, Faculty of Pharmacy and Biotechnology, German University in Cairo, Cairo, Egypt and; 2 Department of Pharmaceutics and Industrial Pharmacy, Faculty of Pharmacy, Ain Shams University, Cairo, Egypt

**Keywords:** Acetazolamide, penetration enhancing, hybridized, vesicles, glaucoma

## Abstract

Usually the topical delivery of ocular drugs poses a great challenge. Accordingly, the work in this study comprised the use of different hybrids of generally regarded as safe (GRAS) oils and surfactants in order to develop and optimize novel acetazolamide (AZD) entrapped-vesicular systems aiming at improving its ocular delivery and reaching better therapeutic outcomes in the treatment of glaucoma. The phospholipid/cholesterol bilayer of the vesicles was enriched with hybrids of Tween 80, Labrasol, Transcutol and Labrafac lipophile WL in different masses and proportions according to a mixture design viz. D-optimal mixture design. Three models were generated comprising three responses: particles size, percentage of entrapment efficiency and amount of drug released after 24 hours (Q24h). The results demonstrated the ability of the penetration enhancing hybrids in modulating the three responses compared to the conventional liposomes. Transmission electron microscope was used to characterize the selected formulations. Sterilization of selected formulations was carried out using gamma radiation and the effect of gamma radiations on entrapment, particle size and *in vitro* release were studied. The selected sterilized formulations were tested *in-vivo* on the eyes of albino rabbits in order to evaluate the efficiency of the novel delivery systems on the intra-ocular pressure reduction (IOP) compared to drug solution and the conventional liposomes. The novel formulations proved their efficiency in reducing the IOP to lower values compared to the conventional liposomes, which pose new successful platform for ocular delivery of AZD and other anti-glaucoma drug analogs.

## Introduction

Ocular drug delivery has always been a challenge for most scientists and formulators due to the complexity of the eye’s anatomy and physiology, which normally protect the eye from toxicants and hinder the penetration of applied ophthalmic drugs to the intended site of action and lead to poor accessibility of drugs to several parts of the eye (Hathout et al., 2007).

The most common known method of ocular drug delivery was still applying drops into the lower cul-de-sac. This place can normally hold from 7 to 9 μl but it can retain up to 20-30 μl if blinking is avoided. But since the volume of the eye drops is usually 50 μl, this usually leads to rapid tear secretion deviating from its normal rate which is 1 μl/min and thus leading to subsequent drainage of the eye drops. As a result, only 1-3% of the applied dose penetrates the cornea and is able to reach the intraocular tissues (Paul et al., 2010; Mishra et al., 2011) and subsequently those applied drops are drained quickly aided by the blinking reflux.

The concentration of the drug in the pre-corneal area is the driving force for its transport by passive diffusion across the cornea and therefore for efficient drug delivery, good corneal penetration and prolonged contact time with the cornea is required. An ideal topical drug formulation enhances the bioavailability by sustaining drug release and remaining in contact with the eye for long time (Rathore & Nema, 2009). In this context, an optimal ocular drug delivery system should be developed so that it could be administered in the form of eye drops, causing no blurred vision or irritability, and would require not more than one or two instillations a day. Much of the published data suggest that in the case of ophthalmic drug delivery systems, an appropriate particle size and a narrow size range ensuring low irritation, adequate bioavailability and compatibility with ocular tissues, should be sought for every administered drug. Accordingly, several micro- and nano-carriers have been adopted for ocular delivery such as the microemulsion (Chan et al., 2008), the vesicular systems such as liposomes (Elbialy et al., 2013), discomes and niosomes (Guinedi et al., 2005), protein-based (Hathout & Omran, 2016) and lipid-based particles and polymeric particles (Pisal et al., 2014).

Acetazolamide (AZD) is still considered as first choice for the treatment of glaucoma (Friedman et al., 1985) and although it is a very effective drug, yet, when used systemically it had several unpleasant side effects, the most common of which are diuresis and metabolic and respiratory acidosis (Galdston, 1955). Other systemic side effects may include gastrointestinal upset, lassitude, paraesthesia, anorexia, weight loss, malasia, depression, decreased libido, blood decrasis and rare complications as renal stones. All these side effects are due to the large distribution of carbonic anhydrase enzyme in the body (Singla et al., 2002).

Due to the many aforementioned side effects of the systemic route, the topical route of AZD is preferred as it leads to decrease in the dose, faster onset of action, decrease in the side effects and consequently improving the patient compliance (Mura et al., 2009). Yet, there are some problems associated with the topical route the main two of them are the poor aqueous solubility of AZD (0.7 mg/ml) and its low permeability coefficient (4.1 × 10^−^^6^) that is insufficient for large amount of the drug to reach the ciliary body.

To this end, the aim of the current work was to develop and optimize novel topical vesicular formulations, which we named penetration enhancing hybridized vesicles that would be able to efficiently deliver AZD topically to the eye. These vesicles are expected to better accommodate and solubilize the drug and increase its penetration capacity compared to the conventional liposomes. Though considered a derivative type of the liposomal vesicles yet these vesicles would differ in both chemical and physiochemical properties. They are also posyulated to show more advantages over the aforementioned conventional counterparts. The estimated main advantage is the vesicular membrane high elasticity thus allowing it to squeeze through pores smaller than their size leading to higher penetration and thus higher bioavailability of the delivered drug (Cevc, 1996; Cevc & Vierl, 2010; Cevc, 2012; Chopra & Cevc, 2014). The deformability mainly depends on the ability of the edge activator or in other words the penetrator enhancer to destabilize the phospholipids packing in the liposomal bilayers (Cevc & Blume, 2001; Cevc et al., 2002). Moreover, the enhancers play a very important role in redistributing the non-uniformally stressed bilayers and facilitating their ability to deliver the targeted drug through the eye layers (Singh et al., 2009). These vesicles were proven to be a safe way of delivering the targeted drug to its site of action (Hughes et al., 2004; Guinedi et al., 2005).

To our knowledge, no work has been previously accomplished in hybridizing and optimizing the presence of several penetration enhancers together in the vesicles phospholipid bilayer membranes and specifically for ocular delivery. In light of the above, we utilized non-ionic surfactants as a source of corneal penetration enhancers such as: Tween 80, Labrasol and Transcutol. Moreover, a non-toxic oil (liquid lipid) such as Labrafac Lipophile WL was also included to impart membrane fluidization in the vesicles phospholipids bilayers leading to more flexibility and elasticity. Only minor and reversible changes in the histology of the ocular tissues were reported for the used surfactants (Manconi et al., 2012).

By improving the penetration and solubilization of the delivered drug, its poor bioavailability is expected to be overcome and hence reducing its frequent administration and minimizing its side-effects would be achieved. The composition of the formulations was established according to the D-optimal mixture design, which is an advanced statistical experimental design that is specific for mixtures and which is based on computational model based on a distance matrix where the determinant of this matrix is optimized (that is the reason why the design is called D-optimal). It was proven to be highly accurate and sufficient to navigate most of the experimental space (Abdel-Hafez et al., 2014). Optimization of vesicular systems using different experimental designs is usually warranted (Soliman et al., 2016).

The formulated vesicles were tested for entrapment efficiency, particle size, *in vitro* release and *in vivo* for their IOP lowering activity and irritancy on the eyes of albino rabbits.

## Materials and methods

### Materials

Acetazolamide was gifted by Chemical Industrial Development Pharmaceutical Company (CID; Cairo, Egypt). Epikuron® 200, which is soya bean phosphatidylcholine was gifted by Cargill (Minneapolis, MN). Transcutol®, Labrasol®, Labrafac Lipophile WL 1349 ® were gifted by Gattefosse (Nanterre, France). Tween 80®, Absolute ethanol and acetone, disodium hydrogen phosphate, potassium dihydrogen phosphate, sodium chloride were purchased from El-Nasr Pharmaceutical Co. (Cairo, Egypt). Cholesterol, chloroform, methanol, PEG 6000, propylene glycol and dialysis membrane with 12 000–14 000 molecular weight cut-off were purchased from Sigma Aldrich (Darmstadt, Germany). Finally, Sodium Thioglycollate medium and Nutrient agar were purchased from Oxoid (Hampshire,UK).

### Methods

#### Preparation of the acetazolamide-loaded penetration enhancing hybridized vesicles

Acetazolamide-loaded penetration enhancing hybridized vesicles (PEHVs) were prepared by the thin film hydration method (Jain et al., 2011). Briefly, the lipid components which are the surfactants and/or oils together with the Epikuron® (phosphatidylcholine) with different ratios were dissolved in a mixture of chloroform: methanol with a ratio 2:1 (v:v), respectively. Consequently, the drug was dissolved in 2 ml acetone and added to the lipid mixture. Then, the used organic solvents were evaporated leaving a thin film of the used lipid using a rotary evaporator (Buchi, Flawil, Switzerland) equipped with an air-pump at a temperature of 53 °C, speed of rotation 90 rpm and pump pressure of 600 bar. After complete dryness of the film, the lipid layer was hydrated, the lipid layer was hydrated using 5 ml PBS and the suspension was rotated by the rotary evaporator at 60 rpm at a temperature of 40 °C for one hour for the vesicles to form at normal atmospheric pressure. Finally, the vesicular suspension was left to cool at room temperature for half an hour and then was kept in a refrigerator at 4 °C. All samples were prepared in triplets.

#### D-optimal mixture design model construction

In order to investigate different effects of the used penetration enhancers for preparing PEHVs composed of soy bean phosphatidylcholine (Epikuron®) together with a blend of surfactants, a D-optimal design mixture design was conducted using the Design-Expert 7.0 software (Stat-Ease Inc, Minneapolis, MN). The demonstrated independent variables were: the individual amounts of each of labrafac lipophile WL, Transcutol, Tween 80 and Labrasol and the dependant variables (responses) were: particle size, entrapment efficiency percentage and the percentage of drug released after 24 h. The points of the design were vertices, centers of edges, axial check blends, interior blends and overall centroid where an overall number of 16 points including three replicate points were generated. Values of the dependant variables; particle size (P.S.), entrapment efficiency (EE%) and the percentage of drug released after 24 h (Q 24 h%), were fed into the design and equations linking the dependant variables with the independent variables were produced. For comparative purpose, two additional formulations were further prepared; conventional liposomes composed of phosphatidylcholine and cholesterol, in addition to vesicles containing neither surfactants nor cholesterol and containing only phosphatidylcholine. The composition of the different penetration enhancing hybridized vesicles (PEHVs) prepared according to the D-optimal mixture design together with the two additional liposomal formulations are shown in the “Supplementary material: Table 1”. All the formulations contained 10 mg of the drug. Three models were conducted, first for the particle size, second for the entrapment efficiency percentage and a final one concerned with the percentage of drug released after 24 h (Q 24 h%).

#### Characterization of the prepared vesicles

##### Determination of the entrapment efficiency percentages (EE%) of AZD

The entrapment efficiency of AZD was determined by measuring the concentration of the un-entrapped amount in the supernatant which was obtained by centrifugation of the vesicular suspension using a cooling centrifuge (Hermle Labortechnik, Wehingen,Germany) at 13 000 rpm for 2 h. The amount of the free drug was determined spectrophotometrically at 264 nm (El-Gazayerly & Hikal, 1997; Guinedi et al., 2005; Hathout et al., 2007) using a UV-visible double beam SpectrophotometerV-530 (Jasco, Gross-Umstadt, Germany). The method was validated where the recorded linearity was *r*^2 ^=^ ^0.9997 (Supplementary material: Figure 1), the accuracy was 99.8% and the limit of quantification was 0.6 μg/ml. Moreover, in order to exclude the effect of other components of the formulations, plain vesicles were prepared and processed typically as the loaded ones and their supernatant was used as a blank during the UV spectrophotometric measurements. The entrapment efficiency was calculated as follows:

$$\mathrm{EE}\%=\left((W_{t}-W_{f}/W_{t})\right)times;100$$

where, *W_t_* represents the total amount of the drug present in the formulation; *W_f_* represents the amount of the free drug found in the supernatant.

##### Particle size and PDI analysis

The particle size and span analysis of all the formulations were measured using laser diffraction analyzer (Mastersizer 2000, Malvern instruments, Worcestershire, UK),where the samples were diluted first using PBS solution to a suitable scattering intensity and redispersed by hand shaking then analyzed (Manconi et al., 2012).

##### *In-vitro* release of AZD from the prepared AZD-loaded penetration enhancing hybridized vesicles

The *in vitro* release study was performed using the dialysis membrane method where the free drug was allowed to be released through a dialysis membrane of 12 000–14 000 molecular weights cut–off, which could retain the prepared vesicles. Briefly, a measured amount of PEHVs containing 2 mg of AZD was transferred to a glass tube of 7 cm length open from two ends and a diameter of 2.5 cm. The lower part was covered with the dialysis membrane, which was previously soaked overnight in PBS solution. The cylinder was fitted on a cover and immersed in a beaker containing 30 ml of PBS containing 1% v/v Tween 80 solution. This amount was calculated based on the sink conditions of the drug. The whole system was maintained at 37 ± 0.5 °C with continuous shaking at 60 rpm for 24 h (GLF Shaker 1038, Burgwedel, Germany). Samples were withdrawn every hour for the first 7 h and then at 24 h. Fresh PBS containing 1% v/v Tween 80 solution was used to replace the withdrawn samples. The samples were assayed spectrophotometrically at 264 nm. All the results were the mean values of three runs.

##### Particle morphology using transmission electron microscopy

Conventional liposomes together with three loaded selected formulations of PEHVs were examined using 1% phosphotungistic acid at 80 kV (Abuhanoglu & Ûzer, 2014) under transmission electron microscope (TEM) (Jem-JEOL, Akishima, Japan) for analyzing the particle size and morphology.

#### Deformability measurement of the optimized PEHVs

The deformability of selected PEHVs formulation was performed using the extrusion method (Khan et al., 2015). Briefly, the penetration enhancing hybridized vesicles were extruded through polycarbonate membrane filter with a pore size of 450 nm (Millipore, Waltham, MA) at a constant pressure of 2.5 bars using a LiposoFast extruder (Avestin^TM^, Manheim, Germany).

The deformability was calculated according to the following equation (Salama et al., 2012):

$$D\mathrm{emsp;}=\mathrm{emsp;}\frac{j}{t}{\left(\frac{\mathrm{rv}}{\mathrm{rp}}\right)}^{2}$$

where *D* is the deformability index (equivalent to the volume of the vesicles passing through the membrane pores in 5 min), *j* is the amount of the vesicles suspension which is extruded (ml), *rv* is the size of vesicles after extrusion (nm) and *rp* is the pore size of the barrier (nm).

#### Sterility studies

##### Sterilization by gamma radiation

Three formulations (F1, F7 and F15) were chosen to be prepared and sterilized.

The aforementioned formulations were selected for different reasons. First, they have different compositions: F1 contains a blend of two penetration enhancers, F7 has only one surfactant; Transcutol while F15 contains a mixture of all the investigated penetration enhancers. Second, they have different particle sizes with low span indices (indicating accurate measurements) and different Q24h values yet high entrapment efficiencies. So, they represent a good variation of the prepared PEHVs that is needed to test the effect of sterilization on these new vesicles.

Each formula was prepared in triplicates then stored in 15 ml falcons. Each sample received irradiation dose of 25 kGy, which was previously proven to be the dose which guarantee full sterilization according to the European pharmacopeia 2006. In addition, this dose was reported not to cause structural changes in the PEHVs similar preparations (Kodaka et al., 2005; Ali & Shafie, 2012). Accordingly, the irradiation facility used at the National Center for Radiation Research and technology (NCRRT) found in the Egyptian atomic Energy Authority was used. It included cobalt-60 gamma chamber 4000-A. Full characterization of the sterilized formulations was performed as explained in the Section “Characterization of the prepared vesicles”.

##### Sterility testing

After applying gamma irradiation, sterility testing was carried out by adopting the pour plate method using thioglycolate medium, which promotes bacterial growth. An aliquot of 1 ml from each formulation was transferred to an agar plate containing the previously mentioned medium. Afterwards, the plates were left for incubation at 37 °C followed by examination to detect any bacterial growth. In addition, positive and negative controls were performed to compare with (Wiegand et al., 2008). In addition, the tube method (Hathout et al., 2007) was performed, where sterilized glass tubes are used which were firmly closed. In each tube, 5 ml of thioglycolate medium was added together with 0.5 ml from each formulation. Positive and negative controls were prepared to compare the results. All samples were incubated at 37 °C and the results were reported after 14 days.

#### *In vivo* studies on the selected AZD-loaded penetration enhancing hybridized vesicles

The efficacy of the selected AZD-loaded PEHVs topical formulations in lowering the IOP was evaluated using normotensive albino rabbits. The results were compared to conventional liposomes and 1% AZD solution. First, all the used preparations were adjusted at a concentration of 1% w/v AZD. Then, albino rabbits of average weight 2–2.5 kg were randomly divided into four groups. Each group contained six rabbits, where group I received 1% AZD solution, group II received AZD-loaded PEHVs formula F7, group III received AZD-loaded PEHVs formula F15 and finally, group IV received AZD-loaded conventional liposomes.

The rabbits were kept in individual cages with food and water and were maintained in a 12-h light/12-h dark cycle in a temperature controlled room at 20–24 °C (Olah et al., 2005; Hathout et al., 2007). A single 100 μl dose of each preparation were administered at room temperature and instilled in the lower conjuctival sac corneal surface of the rabbit’s eye. The IOP was measured as a function of time using a standardized tonometer by the same operator.

The IOP was measured just before drug administration then at 1, 2, 3, 4, 6, 8, 10, 12 and 24 h. In all experimental rabbits, the right eye was left as a control. The ocular hypotensive activity was expressed as the average difference in the IOP between the treated and the control eye in order to eliminate the diurnal, seasonal and individual variations usually observed in rabbits.The experimental procedures were done according to the ethical principles of the Egyptian Research Institute of Ophthalmology (ERIO), Giza (Egypt) on the use of animals and the study was approved by the ethical committee at the German University in Cairo and followed the declaration of Helsinki.

#### Draize test

The main aim of this test was to determine whether the AZD-loaded PEHVs (F7 and F15) and the conventional liposomes F18 being investigated would pose any irritancy to the rabbits eyes as an indication of ocular irritancy on the humans counterparts. For this test, 9 male New Zealand healthy young adult rabbits with age 4-5 month old and weight ranging from 2 to 2.4 kg were used. Animals were examined before use to ensure normal eyes. They were kept in individual cages designed to avoid accidental injury at a temperature of 21 °C. In addition, they were fed with conventional laboratory diets with an unrestricted supply of drinking water. The rabbits were divided into three groups each being a triplicate.

A total of 100 μl of the selected formulations were inserted into the lower conjuctival cul-de-sac of the right eye, while the left eye was kept as a control .The eyelids were held together for several seconds after dose application and normal blinking was allowed. Pre- and post-insertion examination was performed by external observation under proper illumination. Evaluations were performed at 1, 24, 48, 72 h, 7 days, 14 days and 21 days after exposure to the formulation in the form of one drop inserted in the eye daily for one month. Ocular changes were graded by a scoring system that includes rating any alterations to the eye lids, conjunctiva, cornea and iris, pupil reaction or anterior chamber reaction.

## Results and discussion

### Physical characterization of the prepared vesicles and models generation

Table 1 presents the results of [measured entrapment efficiency, particle size, span index and the *in-vitro* percentage drug released after 24 h (Q24h)] of the 16 points that were generated by the D-optimal mixture design together with the results of the conventional liposomes and the vesicles containing phosphatidylcholine only. The D-optimal mixture design was previously proven to be highly accurate and robust in modeling drug carriers based on blends of components such as the microemulsions and lipid nanocapsules (Safwat et al., 2016).

**Table 1.** Characterization of the prepared AZD-loaded penetration enhancing hybridized vesicles.

Formula code	Particle size (μm) ± S.D.	Span index ± S.D.	Entrapment efficiency ± S.D.	Q24h (%) ± S.D.
F1	50.90 ± 0.5	2.12 ± 0.2	93.33 ± 5.25	22.70 ± 0.63
F2	19.74 ± 0.2	9.00 ± 0.5	92.38 ± 0.72	32.00 ± 7.14
F3	7.69 ± 0.1	1.59 ± 0.3	90.13 ± 1.88	33.61 ± 3.99
F4	60.05 ± 0.2	1.76 ± 0.2	92.88 ± 0.54	38.51 ± 2.43
F5	16.93 ± 0.1	7.33 ± 0.4	92.51 ± 0.25	39.12 ± 3.45
F6	13.95 ± 0.1	2.13 ± 0.5	91.92 ± 0.43	48.77 ± 0.26
F7	10.65 ± 0.1	1.44 ± 0.1	91.29 ± 1.58	46.73 ± 2.42
F8	59.19 ± 0.4	1.60 ± 0.1	89.97 ± 4.80	42.14 ± 7.25
F9	10.20 ± 0.4	1.27 ± 0.1	79.53 ± 2.29	34.48 ± 3.84
F10	7.60 ± 0.1	1.32 ± 0.1	92.20 ± 3.26	30.70 ± 2.01
F11	54.65 ± 0.3	2.22 ± 0.3	85.40 ± 1.67	30.75 ± 0.94
F12	57.40 ± 0.5	1.81 ± 0.3	93.97 ± 0.86	40.81 ± 0.69
F13	6.96 ± 0.1	1.32 ± 0.1	91.16 ± 2.12	37.58 ± 12.23
F14	12.57 ± 0.4	1.89 ± 0.2	93.15 ± 1.50	39.39 ± 1.96
F15	56.09 ± 0.3	2.22 ± 0.4	92.39 ± 2.45	33.05 ± 10.54
F16	9.29 ± 0.3	1.22 ± 0.1	92.65 ± 0.43	35.07 ± 1.96
F17	30.15 ± 0.3	14.95 ± 0.4	84.40 ± 1.80	36.20 ± 3.49
F18	19.65 ± 0.2	2.46 ± 0.2	86.80 ± 1.31	25.48 ± 0.79

#### Particle size model

The obtained model was a quadratic one. By applying ANOVA test it was an extremely significant model but with a non-desired significant lack of fit. All the linear mixture components: A, B, C and D besides the terms AB, AD, BD and CD were significant. The other quadratic terms: AC, BC and A^2^, B^2^, C^2^ and D^2^ were non-significant. Accordingly, model reduction was carried out to eliminate the non-significant terms and the ANOVA test was carried out again. This time a significant model was obtained with a desired non-significant lack of fit. In addition, by applying diagnostic tests such as the Box-Cox test, a transformation of the lambda (power) of the response (particle size) was recommended according to the analysis, which calculates the ln of the residuals versus the different power values of the response and the power which corresponds to the lowest ln of residuals is hence warranted (Box & Cox, 1964). Hence, the response was raised to a power of 0.44 and ANOVA was carried again so that another new extremely significant model was obtained with *p* < 0.0001 together with an associated non-significant lack of fit. Accordingly, the modeling of the results was successful using the adopted design (D-optimal mixture design) as demonstrated by the values of *r*^2^ (0.9854), adjusted *r*^2^ (0.9709) and predicted *r*^2^ (Q^2^) (0.60). The high value of adequate precision (19.72) > 4 indicates the sufficiency of the model to navigate the space with the high signal-to-noise ratio of the results.

The particle size model equation obtained is as follows:

(1) $${\left(P.S\right)}^{0.44}\;=+0.13333*\mathrm{Labrasol}+0.15313*\mathrm{Transcutol}+0.12272*\mathrm{Labrafac}\mathrm{Lipophile}\mathrm{WL}+0.29854*\mathrm{Tween}80+0.10564*\mathrm{Labrasol}*\mathrm{Transcutol}-8.05306E-003*\mathrm{Labrasol}*\mathrm{Tween}80-7.51888E-003*\mathrm{Transcutol}*\mathrm{Tween}80-0.014552*\mathrm{Labrafac}\mathrm{lipophile}\mathrm{WL}1349*\mathrm{Tween}80$$

**Figure 1.**
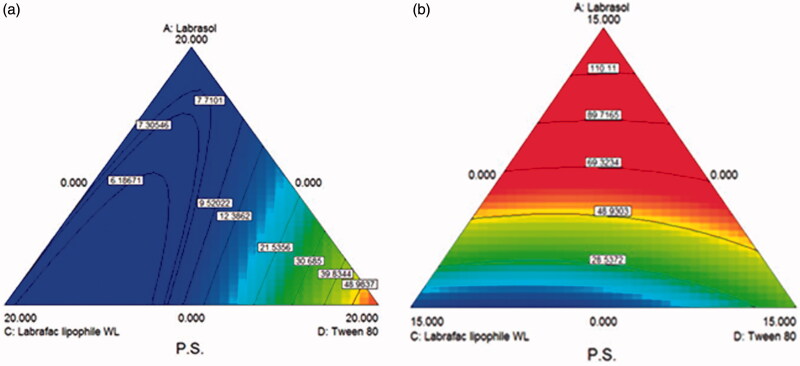
D-optimal mixture design generated contour plots demonstrating the effect of Labrasol, Tween 80 and Labrafac lipophile WL 1349 on the Particle size of the prepared AZD-loaded PEHVs (a) in absence of Transcutol and (b) in presence of 5 mg Transcutol. Moving from red (light) to blue (dark) color indicates increasing particle size.

The obtained contour plots as shown in Figure 1(a) and (b), respectively, confirm the interaction effects of the used oils and surface active agents in decreasing the particle size of the prepared vesicles, where the area of the lowest particle size values lie between labrasol and Labrafac hydrophile WL 1349, while the increase in the percentages of Tween 80 had an increasing effect on the particle size demonstrated by the red area close to the Tween 80 apex in the first figure. It also seems that the presence of Transcutol has an obvious effect on particle size so that when increasing the amount of Transcutol from 0 to 5% the blue areas indicating small particle size decreased. This implies that the presence of Transcutol with the other components increases the particle size of the prepared vesicles. This is attributed to the fact that Tween 80 and Transcutol are more hydrophilic molecules and it was proven that the presence of hydrophilic molecules at the interface lead to increase in the particle size due to the increase in the surface energy, which leads to vesicle enlargement (Mura et al., 2009). The significant effect of these two compounds is also obvious in the high coefficients of Tween 80 and Transcutol factors in the model equation as compared to Labrasol and Labrafac lipophile WL 1349 counterparts.

#### Entrapment efficiency model

The ANOVA test was performed to analyze the results of the entrapment efficiencies where a significant quadratic model was obtained with a desired non-significant lack of fit. The linear mixture terms: A, B, C and D were significant in addition to the interaction term AD. Hence, for further optimization, model reduction was adopted eliminating the other quadratic non-significant terms. The model obtained was an extremely significant one with a non-significant lack of fit. The *r*^2^ was found to be 0.90 with an adjusted *r*^2 ^=^ ^0.84 and predicted *r*^2^ with reasonable agreement with the adjusted *r*^2^ and equals 0.74. The adequate precision was 14.62; a value much higher than 4 indicating a high signal–to-noise ratio and the excellent ability of the obtained model to navigate the space (Abdel-Hafez et al., 2014; Mehanny et al., 2016). A Box-Cox evaluation was performed which revealed no need for further transformation of the power (lambda) of the response (the entrapment efficiency percentage).

The model equation for entrapment efficiency percentage obtained is as follows:

(2) EE% =+3.97305*Labrasol+4.62830*Transcutol+4.57000*LabrafaclipophileWL+4.61728*Tween80+0.068482*Labrasol*Tween80

**Figure 2.**
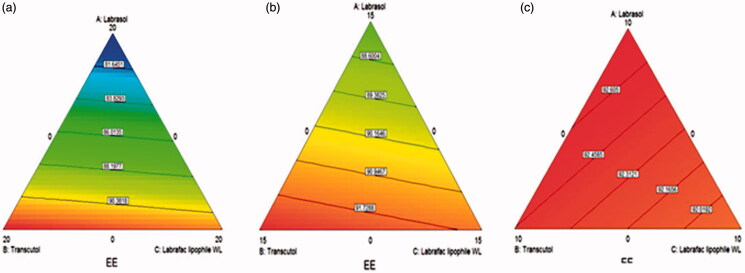
D-optimal mixture design generated contour plots demonstrating the effect of Labrasol, Transcutol and Labrafac lipophile WL 1349 on the entrapment efficiencies of the prepared AZD-loaded PEHVs (a) in absence of Tween 80, (b) in presence of 5 mg Tween 80 and (c) in presence of 10 mg Tween 80. Moving from red (light) to blue (dark) color indicates increasing entrapment efficiencies.

The contour plots obtained shown in Figure 2(a), (b) and (c) showed the effect of the interaction between the mixture components on the entrapment efficiency percentage. A case which is highly clear with the increasing amounts of Tween 80 in the mixture shifting the entrapment values to higher values moving from 0 to 5 to 10% as demonstrated by the increased red areas in the plots indicating higher entrapment efficiencies values.

#### *In-vitro* release and model

**Figure 3.**
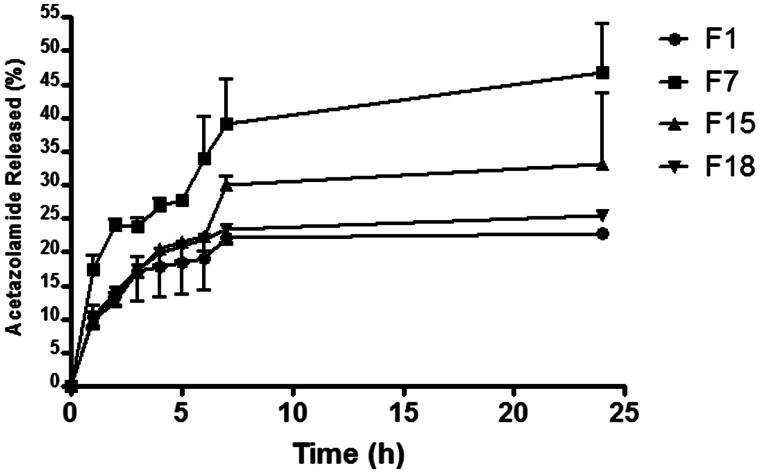
Release patterns of AZD from selected penetration enhancing hybridized vesicles.

Figure 3 demonstrates the release pattern of AZD from different selected formulations (containing different components and having different particles size and entrapment efficiencies in comparison with the conventional liposomes). The sustained release profile was obvious throughout the different combinations. Compared to the conventional liposomes, the release of F7 containing Transcutol only scored the highest percentages released while maintaining the sustained profile, which can be explained by the small particle size of these vesicles with higher available surface areas available for drug release. Formula F15 which contained a blend of all the investigated penetration enhancers in equal amounts followed F7 regarding the amounts released. This may be attributed to the presence of the surfactants and/or oils that fluidize the phospholipid bilayer (Cevc, 1996) of the vesicles introducing defects in its organizational architecture leading to faster drug leakage. F1 demonstrated similar profile with close percentage released values to the conventional liposomes, which may be ascribed to its large measured particle size imparting lower particles surface area available for drug release.

The percentages of drug released after 24 h (Q24) were chosen as a criteria for comparison between the different prepared vesicles. By applying the ANOVA test to analyze these *in-vitro* release results, a quadratic extremely significant model was obtained that was characterized by a desired non-significant lack of fit.

All the linear mixture components: A, B, C and D besides the term BD were significant. The other quadratic terms: AB, AC, AD, BC, CD and A^2^, B^2^, C^2^ and D^2^ were non-significant. Therefore, a model reduction was carried out to eliminate the non-significant terms and the ANOVA test was carried out again which resulted in *p* = 0.0002.

The modeling of the results was successful using the adopted design (D-optimal mixture design) as demonstrated by the values of *r*^2^ (approximately 0.90), the adjusted *r*^2^ (0.81) and the predicted *r*^2^ (Q^2^) (0.60). Again, the high value of adequate precision (11.82) > 4 indicated the sufficiency of the model to navigate the space. A Box-Cox plot was carried out again, which demonstrated the location of the used lambda in close proximity to the best recommended lambda (1.41) and lying within the confidence intervals (-2.31 to 4.43) indicating no recommended further transformation.

Hence, the obtained model equation is as follows:

(3) $${\left(Q24\right)}^{2\;}=+50.43165*\mathrm{Labrasol}+110.71625*\mathrm{Transcutol}+56.23341*\mathrm{Labrafac}\mathrm{lipophile}\mathrm{WL}+84.91793*\mathrm{Tween}80-10.09951*\mathrm{Transcutol}*\mathrm{Tween}80$$

**Figure 4.**
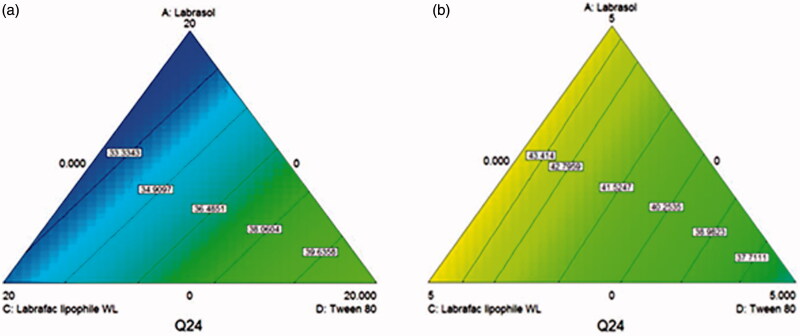
D-optimal mixture design generated contour plots showing the effect of Labrasol, labrafac lipophile Wl 1349 and Tween 80 on the percentage drug released after 24 h of AZD from the prepared penetration enhancing hybridized vesicles (a) in absence of Transcutol and (b) in the presence of 15 mg Transcutol. Moving from blue (dark) to lighter colors indicate higher percentages released.

The obtained contour plots as shown in Figure 4(a) and (b) confirmed the interaction effects of the used oils and surface active agents on the release of AZD from the prepared penetration enhancing hybridized vesicles, where in absence of Transcutol, the amount released after 24 h increased with increasing the percentages of Tween 80 in the surfactants mixture, while in presence of 15 mg Transcutol the amount released increased significantly in the opposite direction (increasing labrasol and labrafac lipophile WL 1349) as demonstrated by increasing the blue and green areas. Transcutol has a shorter chain (Manconi et al., 2012) than the other investigated surfactants. Therefore, its presence suggests the introduction of defects and fluidity in the vesicles facilitating the drug diffusion. Besides, the high compatibility and interaction of Transcutol with Labrasol and labrafac lipophille WL (Mura et al., 2009; Manconi et al., 2012) leads to more membrane fluidization and higher release percentages.

### Visualizing the morphology of the prepared AZD-loaded penetration enhancing hybridized vesicles using transmission electron microscopy

**Figure 5.**
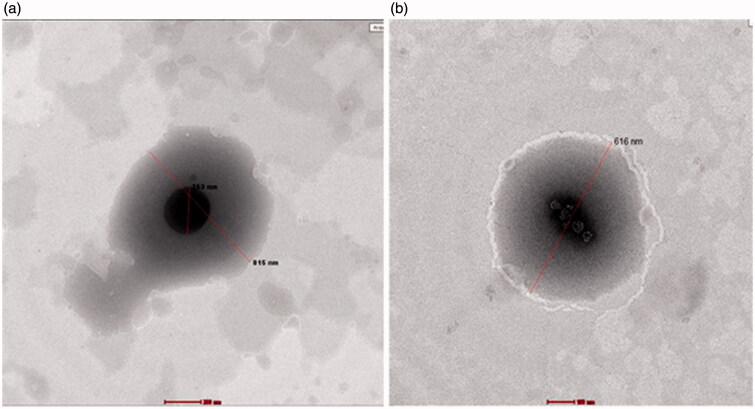
TEM images for aAZD-loaded penetration enhancing hybridized vesicles (a) F1 and (b) F7.

Figure 5 demonstrates the morphological appearance of selected penetration enhancing vesicles (F1and F7) as imaged by transmission electron microscopy and stained using 1% phosphotungistic acid. The spherical morphology of the vesicles is clear. Besides, the small hydrophilic core (darker in color) is revealed. It is known that phosphotungistic acid is a hydrophilic dye that is usually concentrated in aqueous domains **(**Hathout et al., 2007; Mura et al., 2009; Shen, 2010**)**. The lighter colored area represents the multilamellar hydrophobic vesicles bilayer. The smaller obtained particle size in the TEM images is mainly attributed to the drying step during samples preparation that usually causes particles shrinkage and some artifacts (Fagir et al., 2015). Moreover, the TEM images are not reliable for particle size measurements because the photographed image may fall away from the mean of the particle size and may reside within the extremities of the particle size distribution. In order to be reliable, a minimum of 100 images should be taken.

### Deformability study

The performed deformability study has proven the elasticity of the selected formulation (F7) containing Transcutol in the phospholipid membrane bilayer, where these penetration enhancing hybridized vesicles could squeeze themselves and pass through a smaller sized membrane and subsequently scoring a deformability index of 24 ± 2.16 compared to the conventional liposomes, which previously scored 6.4 ± 0.5 (Mishra et al., 2007).

### Sterilization of the selected PEHVs

#### Sterility testing

Sterility testing was performed on the prepared acetazolamide loaded penetration enhancing hybridized vesicles after gamma sterilization using 25 kGy which was proven to be the recommended irradiation dose for the pharmaceutical preparations (Youshia, 2012). Sterility testing using tube method and in addition to the pour plate method were used to ensure the sterilization efficacy. After 14 days, no bacterial growth was observed for both methods confirming the efficacy of the irradiation process.

#### Effect of sterilization on the particle size of selected AZD-loaded penetration enhancing hybridized formulations

The effect of sterilization on the integrity and physical properties of the prepared vesicles was evaluated by applying paired *t*-test between the particle size and span index before and after sterilization. The obtained *p* values were equivalent to *p* = 0.9853 and *p* = 0.1920, respectively, indicating no significant differences between these two parameters before and after sterilization (Supplementary material: Figure 2), which ensures the safe usage of gamma sterilization on these vesicles.

#### Effect of sterilization on the entrapment efficiency and the amount of drug released after 24 h of selected AZD-loaded penetration enhancing hybridized vesicles formulations

By applying paired *t-*test for the entrapment efficiency and the percentage of drug released after 24 h (Q24h, %), the obtained *p* value was equal to 0.1402 and 0.2237, respectively, indicating no significant differences before and after applying sterilization utilizing gamma radiations (Supplementary material: Table 2) and hence the safe use of this method for the sterilization of this new type of vesicles; PEHVs.

### *In-vivo* testing of selected AZD-loaded PEHVs on rabbits

Formula F7 was chosen since it showed a small particle size (10.65 μm), high drug entrapment efficiency (91.29%) and high release values equivalent to 46.73% maintaining the sustained release profile compared to the conventional liposomes. Moreover, adequate morphological appearance indicating the integrity of structure was also obtained for this formulation. Furthermore, it retained its stability after gamma radiation sterilization and after storage for one month with an entrapment value equivalent to 90.50%. In addition to F7, Formula F15 was also evaluated in order to investigate the effect of a hybrid type of PEHVs (containing several penetration enhancers).

**Figure 6.**
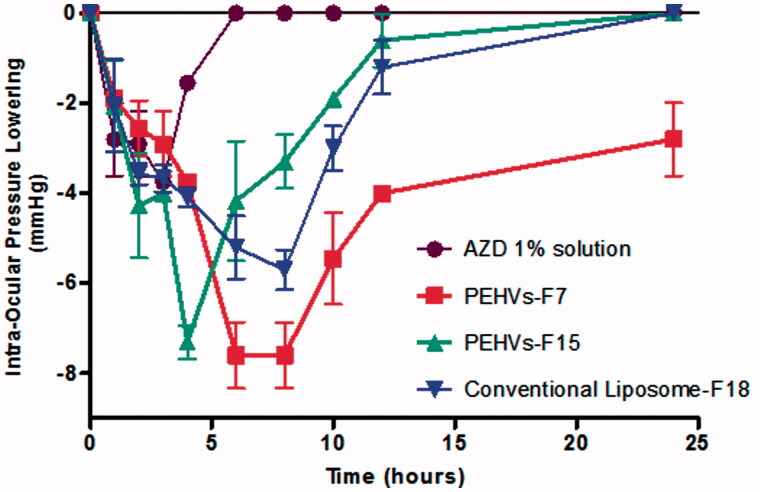
IOP lowering effect of selected AZD-loaded penetration enhancing hybridized vesicles compared to 1% AZD solution and conventional liposomes (Mean ± SEM).

Figure 6 shows the reduction in the IOP of the selected PEHVs compared to the conventional Liposomes and 1% AZD solution. As being observed, 1% AZD solution led to reduction in the IOP effect after 3 h, when a value of −3.75 mmHg was recorded and lasted for a total of 4 h. On the other hand, the IOP lowering effect of formula PEHVs-F7 reached its peak after 6 h recording −7.6 mmHg with an effect that lasted for 24 h thus showing the largest reduction and sustained effect, while the IOP lowering effect of PEHVs -F15 reached its peak after 4 h recording −7.3 mmHg and lasting for 12 h only. Finally, the conventional liposomes (F18) showed reduction in the IOP after 6 h reaching −5.73 mmHg and lasting for 12 h only.

The superiority of F7 was also reflected in calculating the AUC using GraphPad Prism® v.5.0 (Stat-Ease Inc., Minneapolis, MN), where it scored the highest value indicating the highest pharmacological response (*p* < 0.05), followed by F15 and conventional liposomes (no significant differences were recorded between these later formulations) then finally the 1% AZD solution possessing the least AUC (Table 2).

**Table 2.** Comparison between the *in vivo* efficacy of acetazolamide PEHVs compared to 1% acetazolamide solution and conventional liposomes.

Acetazolamide formulation	Maximum value of lowering IOP (mmHg) ± S.D.	Duration of action (lowering IOP) (h)	Area under the curve (AUC) (mmHg.h) ± S.D.
Acetazolamide 1% solution	−3.75 ± 0.07	4	11.78 ± 1.54
PEHVs-F7	−7.6 ± 1.28	>24 hrs	99.08 ± 20.1
PEHVs-F15	−7.3 ± 0.65	12	44.22 ± 10.38
Conventional liposomes-F18	−5.7 ± 0.77	12	51.65 ± 10.77

The previous results showed that entrapping the AZD in PEHVs showed significant enhancement in the lowering of the IOP and an increase in its duration compared to AZD solution. This augmented IOP lowering activity can be ascribed to the improvement in the penetration of AZD entrapped in PEHVs (Youshia, 2012).

A significant IOP reduction associated with a sustained effect was observed in case of PEHVs-F7. This can be justified according to the nature of Transcutol®. Since this surfactant has a short carbon chain, therefore, it enhances the fluidity of the vesicles membrane allowing higher deformability as proven in the adopted deformability study leading to better corneal penetration and also a higher release of the drug (Manconi et al., 2012). This is obvious if compared to the conventional liposomes, which contain embedded cholesterol in its membrane rendering it highly rigid and causing a hindrance of the drug release. This is reflected in its lower IOP lowering activity (Singh et al., 2009).

### *In-vivo* irritancy testing of selected AZD-loaded PEHVs on rabbits eyes using Draize test

All the rabbits being examined over a period of 21 days showed no changes in the iris, cornea, pupil region and the anterior chamber region. However, mild swelling of grade (+1) for the conjunctiva and mild chemosis of grade (+1) were observed during the first hour in case of F15, which disappeared at the next examination period (24 h). On the other hand, in case of F7 and F15 no changes were observed (Supplementary Material: Table 3). These results propose the safe use of PEHVs in the ocular delivery of drugs.

## Conclusion and future perspectives

In this work, hybrids of lipids and surfactants (penetration enhancers) were used and optimized using the highly robust and accurate D-optimal mixture statistical design in order to enhance the physicochemical and biological properties of the conventional liposomes. The prepared PEHVs were proven to possess all the needed characteristics such as suitable particle size, high entrapment efficiency, sustainment of drug release, good stability, sterilization endurance, high drug bioavailability and lack of irritancy to pose a successful delivery system of an anti-glaucoma drug viz. AZD. The useful projection of the use of the studied new vesicles into the delivery of other ocular drugs is thus recommended.

## Supplementary Material

Supplementary_Material.docx

## Notes

**Supplementary material available online.**
