# Supplementary material for: Optimizing novel penetration enhancing hybridized vesicles for augmenting the in-vivo effect of an anti-glaucoma drug
Source: Drug Deliv. 2017 Feb 3;24(1):99–108. doi: 10.1080/10717544.2016.1233588 (PMC8248826; doi:10.1080/10717544.2016.1233588)

**Supplementary Material**

***Optimizing Novel Penetration Enhancing Hybridized Vesicles for Augmenting the In-vivo Effect of an***

***Anti-Glaucoma Drug***

**Sarah S. Naguib^1^, Rania M. Hathout^1, 2*^, Samar Mansour^1,2^**

^1^Department of Pharmaceutical Technology, Faculty of Pharmacy and Biotechnology, German University in Cairo, Cairo, Egypt

^2^Department of Pharmaceutics and Industrial Pharmacy, Faculty of Pharmacy, Ain Shams University, Cairo, Egypt

^*^Correspondence:

Rania M. Hathout, Department of Pharmaceutics and Industrial Pharmacy, Faculty of Pharmacy, Ain Shams University, African Union Organization Street, Abbassia, Cairo, Egypt.

P.O. Box: 11566

Tel: +2 (0) 100 5254919 / + 2 (02) 22912685

Fax: +2 02 24011507

E-mail: r_hathout@yahoo.com

Supplementary Material: Table (1): Composition of the prepared acetazolamide loaded Penetration Enhancing Hybridized Vesicles formulations

| **Formula**  **Code** | **Soya bean phosphatidylcholine (mg)** | **Cholesterol**  **(mg)** | **Labrasol (mg)** | **Transcutol (mg)** | **Labrafac Lipophile (mg)** | **Tween 80**  **(mg)** |
| --- | --- | --- | --- | --- | --- | --- |
| **F 1** | 80 | 0 | 10 | 10 | 0 | 0 |
| **F 2** | 80 | 0 | 0 | 10 | 0 | 10 |
| **F 3** | 80 | 0 | 0 | 0 | 20 | 0 |
| **F 4** | 80 | 0 | 2.5 | 12.5 | 2.5 | 2.5 |
| **F 5** | 80 | 0 | 10 | 0 | 0 | 10 |
| **F 6** | 80 | 0 | 0 | 20 | 0 | 0 |
| **F 7** | 80 | 0 | 0 | 20 | 0 | 0 |
| **F 8** | 80 | 0 | 0 | 0 | 0 | 20 |
| **F 9** | 80 | 0 | 20 | 0 | 0 | 0 |
| **F 10** | 80 | 0 | 10 | 0 | 10 | 0 |
| **F 11** | 80 | 0 | 12.5 | 2.5 | 2.5 | 2.5 |
| **F 12** | 80 | 0 | 0 | 0 | 0 | 20 |
| **F 13** | 80 | 0 | 0 | 0 | 20 | 0 |
| **F 14** | 80 | 0 | 0 | 10 | 10 | 0 |
| **F 15** | 80 | 0 | 5 | 5 | 5 | 5 |
| **F 16** | 80 | 0 | 0 | 0 | 10 | 10 |
| **F 17** | 100 | 0 | 0 | 0 | 0 | 0 |
| **F 18** | 80 | 20 | 0 | 0 | 0 | 0 |

^*^All formulations contained **10 mg** Acetazolamide

Supplementary Material: Table (2): The Effect of sterilization on the Entrapment Efficiency and *In-vitro* release after 24 h of selected acetazolamide loaded Penetration Enhancing Hybridized vesicles formulations.

| **Formula code** | **Before Sterilization** | | **After Sterilization** | |
| --- | --- | --- | --- | --- |
|  | **EE% ± S.D.** | **Q24h (%)**  **± S.D.** | **EE% ± S.D.** | **Q24h (%)**  **± S.D.** |
| **F1** | 93.33 ± 5.25 | 22.70 ± 0.63 | 87.14 ± 1.21 | 22.77 ± 0.092 |
| **F7** | 91.29 ±1.58 | 46.73 ± 2.41 | 90.45 ± 7.24 | 34.17 ± 7.71 |
| **F15** | 92.39 ± 2.45 | 33.05 ± 10.53 | 84.93 ± 6.65 | 26.48 ± 2.99 |

**Supplementary Material: Table (3): Draize test scoring results for AZD loaded PEHVs (F7 , F15) compared to the conventional liposomes (F18**).

| **F18** | **F18** | **F18** | **F15** | **F15** | **F15** | **F7** | **F7** | **F7** | **Time/group** |
| --- | --- | --- | --- | --- | --- | --- | --- | --- | --- |
| 0 | 0 | 0 | 0 | +1chemosis | +1conj | 0 | 0 | 0 | **1h** |
| 0 | 0 | 0 | 0 | 0 | 0 | 0 | 0 | 0 | **24h** |
| 0 | 0 | 0 | 0 | 0 | 0 | 0 | 0 | 0 | **48h** |
| 0 | 0 | 0 | 0 | 0 | 0 | 0 | 0 | 0 | **72h** |
| 0 | 0 | 0 | 0 | 0 | 0 | 0 | 0 | 0 | **7day** |
| 0 | 0 | 0 | 0 | 0 | 0 | 0 | 0 | 0 | **14day** |
| 0 | 0 | 0 | 0 | 0 | 0 | 0 | 0 | 0 | **21day** |

Supplementary Material: Figure (1): Calibration curve of acetazolamide in PBS (pH = 7.4) using UV spectrophotometric measurement at 264 nm. Points represent Mean ± SD (Too low values so neglected).

Supplementary Material: Figure (2) (a) Particle size and (b) Span index of the selected acetazolamide loaded Penetration Enhancing Hybridized vesicles formulations before and after sterilization using gamma radiation.


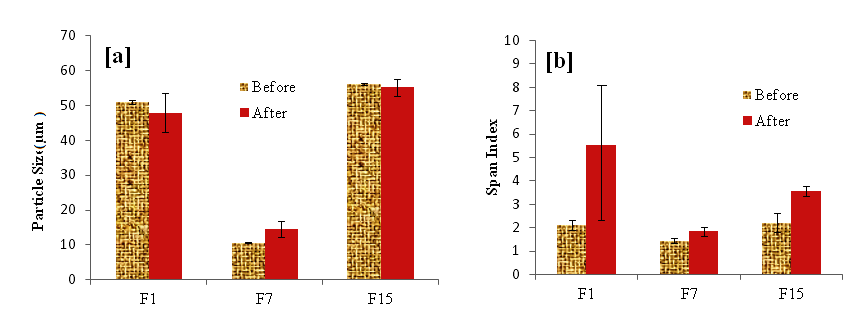

Supplement: Supplementary_Material.docx [file IDRD_A_1233588_SM8422.docx]
